# Integration of mechanical and chemical signals by YAP and TAZ transcription coactivators

**DOI:** 10.1186/2045-3701-3-33

**Published:** 2013-08-28

**Authors:** Xiaocan Guo, Bin Zhao

**Affiliations:** 1 Life Sciences Institute, Zhejiang University, Hangzhou, Zhejiang 310058, China

**Keywords:** YAP, TAZ, The Hippo pathway, mechanical stress, GPCR signaling, Wnt pathway

## Abstract

YAP and TAZ are transcription coactivators and effectors of the Hippo pathway, which play a key role in organ size control. Through interaction with transcription factors such as TEADs, they activate gene transcription and thus promote cell proliferation, inhibit apoptosis, and regulate cell differentiation. Dysregulation of YAP/TAZ was found to correlate with human cancers. The oncogenic roles of these proteins were also demonstrated in animal models. The growth promoting activity of YAP/TAZ is limited by the Hippo tumor suppressor pathway through phosphorylation-induced cytoplasmic retention and destabilization. Recently, it was found that YAP and TAZ mediate responses to several extracellular signals including mechanical stress, GPCR signaling, and the Wnt signaling pathway. All these growth-regulating signals play important roles in normal development and cancer. In this review, we would like to discuss the function of YAP and TAZ as effectors of these physiological signals.

## Introduction

The precise control of cell number during development and regeneration maintains organ size homeostasis of multicellular organisms. In recent years, the Hippo signaling pathway is emerging as a key regulator of organ size [1,2]. The Hippo pathway was first identified in *Drosophila* by genetic mosaic screens for growth inhibitory genes [3-12]. Then the pathway and its function in organ size control were found to be conserved in mammals [13,14]. Yes-associated protein (YAP) and its homolog transcriptional co-activator with PDZ-binding motif (TAZ, also called WWTR1) are key downstream effectors of the Hippo pathway being phosphorylated and inhibited by the Hippo pathway kinases Last1/2 [13,15]. YAP and TAZ activate gene transcription through interaction with transcription factors such as the four TEAD family proteins [16,17]. YAP/TAZ-induced gene expression results in cell proliferation, evasion of apoptosis, and also amplification of progenitor/stem cells thus promotion of organ size. Consistently, recent studies on animal models also demonstrate a role of the Hippo pathway and YAP in tissue regeneration [18,19]. Furthermore, there are ever-accumulating reports on the correlation of abnormal YAP/TAZ activation with human cancers [20-23]. However, upstream signals regulating the Hippo pathway and YAP/TAZ were obscure until the publication of several recent studies [24-28]. In this review we discuss the roles of YAP/TAZ as mediators of responses to mechanical stress, GPCR signaling and the Wnt signaling.

### Biochemical and biological functions of YAP/TAZ

YAP was first cloned as a protein bound to non-receptor tyrosine kinase YES1 [29]. YAP mRNA is ubiquitously expressed in a wide range of tissues, except peripheral blood leukocytes [30]. There are two major splicing variants with one (YAP1) or two (YAP2) WW domains. The function of YAP remained enigmatic until it was shown to be a transcription co-activator [31]. A reporter assay demonstrated that the C-terminal region of YAP has strong transcriptional activation activity. However, it does not directly bind to DNA. Instead, it is brought to gene promoters through the interaction with transcription factors. First identified by affinity purification [32], TEAD family transcription factors are major partners of YAP in regulation of cell proliferation and organ size [17]. The importance of TEADs in the function of YAP was nicely demonstrated by a mouse model with knock-in of a mutant YAP deficient in interaction with TEAD. Remarkably, the knock-in mice showed skin phenotypes closely resembled that of the YAP knock-out mice [33]. Furthermore, a heterozygous YAP-binding-deficient mutation of TEAD1 leads to a human genetic disease Sveinsson’s chorioretinal atrophy [17,34,35], which further illustrates the physiological importance of TEAD in YAP function. Through the centrally localized WW domains, YAP also interacts with other transcription factors such as RUNX1/2 and Smad1 [31,36], which may also contribute to YAP-induced gene expression and organ size control. The mechanism by which YAP activates transcription is still elusive. However, the *Drosophila* homolog of YAP, Yki, was shown to interact with GAGA factor (GAF), the Brahma complex, and the Mediator complex in cell nuclei to activate gene expression [37]. Interestingly, another report demonstrated that Scalloped (Sd), the *Drosophila* homolog of TEADs, function as a default transcription repressor by binding to Tgi [38]. When activated, Yki competes with Tgi for Sd binding and thus switches Sd from OFF to an ON state. Importantly, the function of Tgi is conserved in its mammalian homolog Vestigial-like 4 (Vgl4) [38].

YAP has an evolutionarily conserved function in organ size control. For instance, liver-specific overexpression of YAP in transgenic mice results in enlarged liver, which is reversible upon cessation of YAP overexpression [14,39]. However, sustained YAP overexpression eventually leads to the development of liver tumors [14]. Examination of clinical samples identified genomic amplification as well as elevated expression and nuclear localization of YAP in human cancers [13,14,20,21,40]. Further experiments on cultured cells supported the function of YAP in inducing cell transformation, loss of cell-contact-inhibition, and epithelial-mesenchymal transition (EMT) [13,41]. Thus current findings indicate an oncogenic role of YAP. YAP activation also promotes the expansion of tissue-specific progenitor cells such as in skin, chicken neural tube, and more controversially in intestine, which would be discussed below [39,42-44]. Furthermore, YAP promotes self-renewal of mouse ES cells and represses differentiation [45]. Taken together, YAP has important functions in organ size control, tumorigenesis and tissue regeneration.

TAZ is a YAP paralog initially identified as a 14-3-3 binding protein [46]. In human and mouse, TAZ mRNA is expressed in all tissues except thymus and peripheral blood leukocytes, with the highest expression in kidney [46]. TAZ has approximately 50% sequence identity and very similar topology with YAP. Clued by the transcription coactivator function of YAP, TAZ was also confirmed to have similar activity highly dependent on TEAD family transcription factors [16,46]. However, some target transcription factors are unique to TAZ, such as Smad2/3 and Pax3, which may contribute to the differential functions of TAZ to YAP [47,48]. TAZ also promotes cell proliferation, induces EMT, increases cell migration and invasion in vitro [15,22], and is shown to be overexpressed in approximately 20% of breast cancer samples [22]. Interestingly, a recent study identified TAZ as a key factor sustaining self-renewal of breast cancer stem cells [23]. Despite the functional similarity of YAP and TAZ, they still possess some clearly distinct characters. For instance, YAP and TAZ knockout mice show different phenotypes: YAP knockout animals are embryonic lethal and show shortened body axis and defects in yolk sac vasculogenesis [49]. In contrast, TAZ knockout mice are viable and are characterized by renal cysts which lead to end stage kidney disease [50,51]. In addition, in many cases, the phenotype of YAP or TAZ knockdown were not compensated by the presence of the other [17,22,47,52]. Such differences may be explained by spatial and temporal regulation of YAP and TAZ activity or different downstream targets, which require further study.

### Mechanisms regulating YAP/TAZ activity

YAP and TAZ regulate gene expression. However, the regulation on transcription of themselves is largely unknown. Recently it was reported that an Ets family transcription factor GABP directly promotes YAP transcription under inhibition by oxidative stress [53]. However, most of the known mechanisms regulating YAP/TAZ activity are on post-translational level. The physiological significance of YAP/TAZ was first revealed after the identification of *Drosophila* Yki as a key effector of the Hippo pathway [54]. Detailed biochemical analysis indicated that YAP is directly phosphorylated by Lats1/2 on five consensus HXRXXS motifs [13-15,55,56]. Phosphorylation of S127 in YAP promotes 14-3-3 binding, resulting in cytoplasmic sequestration and therefore inactivation of YAP [13-15,55,56]. Phosphorylation on YAP S381 primes subsequent phosphorylation by another kinase, possibly casein kinase 1 (CK1δ/ϵ), thereby activates a phosphodegron degradation motif. Subsequently, the activated phosphodegron recruits the E3 ubiquitin ligase SCF^β-TRCP^, leading to poly-ubiquitination and degradation of YAP [57]. The importance of YAP as a downstream effector of the Hippo pathway was elegantly demonstrated in vivo by the reversal of Hippo pathway deficiency-induced oncogenic phenotypes by loss of one allele of YAP [58,59]. TAZ has four conserved Lats1/2 target motifs and is regulated by the Hippo pathway in a similar manner [15,60].

Besides the canonical Hippo pathway, YAP/TAZ are also regulated by physical interactions with other proteins, especially cell junctional proteins. Through the WW domains, YAP could interact with angiomotin (AMOT) family proteins, which results in YAP localization to tight junction and YAP inhibition through phosphorylation-dependent and -independent mechanisms [61-63]. YAP and TAZ also interact with another tight junction protein ZO-2, which was reported to increase nuclear localization of YAP and tight-junction localization of TAZ, respectively [64,65]. Interestingly, a major adherens junction protein alpha-catenin, can also bind to and inhibit YAP by mediating its cell-cell junction and cytoplasmic localizations [33,44]. Another adherens junction protein PTPN14 has also been reported by several groups to be a negative regulator of YAP through the interaction with YAP WW domains [66-69]. In consistence with these finding on YAP/TAZ regulation by junctional proteins, YAP/TAZ were found to be regulated by cell-cell contact. In tissue culture, high cell density induces YAP phosphorylation and cytoplasmic translocation [13] and disruption of cell-cell junctions results in the nuclear localization of YAP/TAZ [70]. In mouse blastocysts, YAP is nuclear in outer layer cells, and cytoplasmic in the inner blastocyst layer cells [71]. Taken together, it is clear that YAP/TAZ transcription coactivators might mediate upstream signals through both the Hippo pathway and their interactions with other proteins.

### YAP/TAZ mediate cellular responses to mechanical stress

Biomechanics is increasingly recognized as an important regulator of cell physiology and a key player in development and pathological abnormalities. For instance, many cancers such as breast cancer have elevated tissue stiffness due to altered ECM composition. Remarkably, softening of the tumor microenvironment slows tumor growth and progression [72]. It is also known that matrix stiffness is a determinant factor for lineage commitment of mesenchymal stem cells (MSCs). MSCs differentiate into adipocytes on soft matrix whereas osteoblasts on stiff matrix [73]. However, little was known about the molecular mechanisms transducing these conditions into the nucleus and resulting in physiological response. It was known that TAZ could promote osteogenesis and repress adipogenesis of MSCs possibly through activation of RUNX2-dependent gene transcription and inhibition of PPARγ-dependent gene expression [52]. This raises an interesting possibility of YAP/TAZ in mediating MSC differentiation in response to mechanical stress. This speculation was recently proved to be true [25]. When cells were grown on soft matrix or on micropatterned small islands, cells adopted a round shape and YAP/TAZ were mostly cytoplasmic. However, when cells were grown on stiff materials or large adhesive islands, they became nuclear and thus active. More importantly, the activity of YAP and TAZ determines the lineage commitment of MSCs in response to matrix stiffness. Cell adhesion and suspension are two conditions affect cell geometry in an analogous but more potent way than different matrix stiffness. It was found that YAP/TAZ subcellular localization is regulated by cell adhesion/suspension in a way similar to matrix stiffness [74]. Thus, YAP and TAZ are key nuclear effectors of mechanical stress.

Contraction of the actomyosin cytoskeleton plays a central role in generation and transducing mechanical forces in cells. Consistently, the regulation of YAP/TAZ localization by mechanical stress depends on F-actin and Rho family GTPases [25,74,75]. Disruption of F-actin or inhibition of Rho by specific inhibitors inactivates YAP. On the contrary, induced actin polymerization by overexpression of F-actin nucleator diaphanous correlates with activation of YAP/TAZ [76]. Regulation of the *Drosophila* Yki by F-actin has also been demonstrated in vivo [76]. Disruption of F-actin in vivo through several different genetic manipulations results in Yki activation and overgrowth of *Drosophila* tissue. Importantly, Yki was found to be required for the overgrowth.

**Figure 1:**
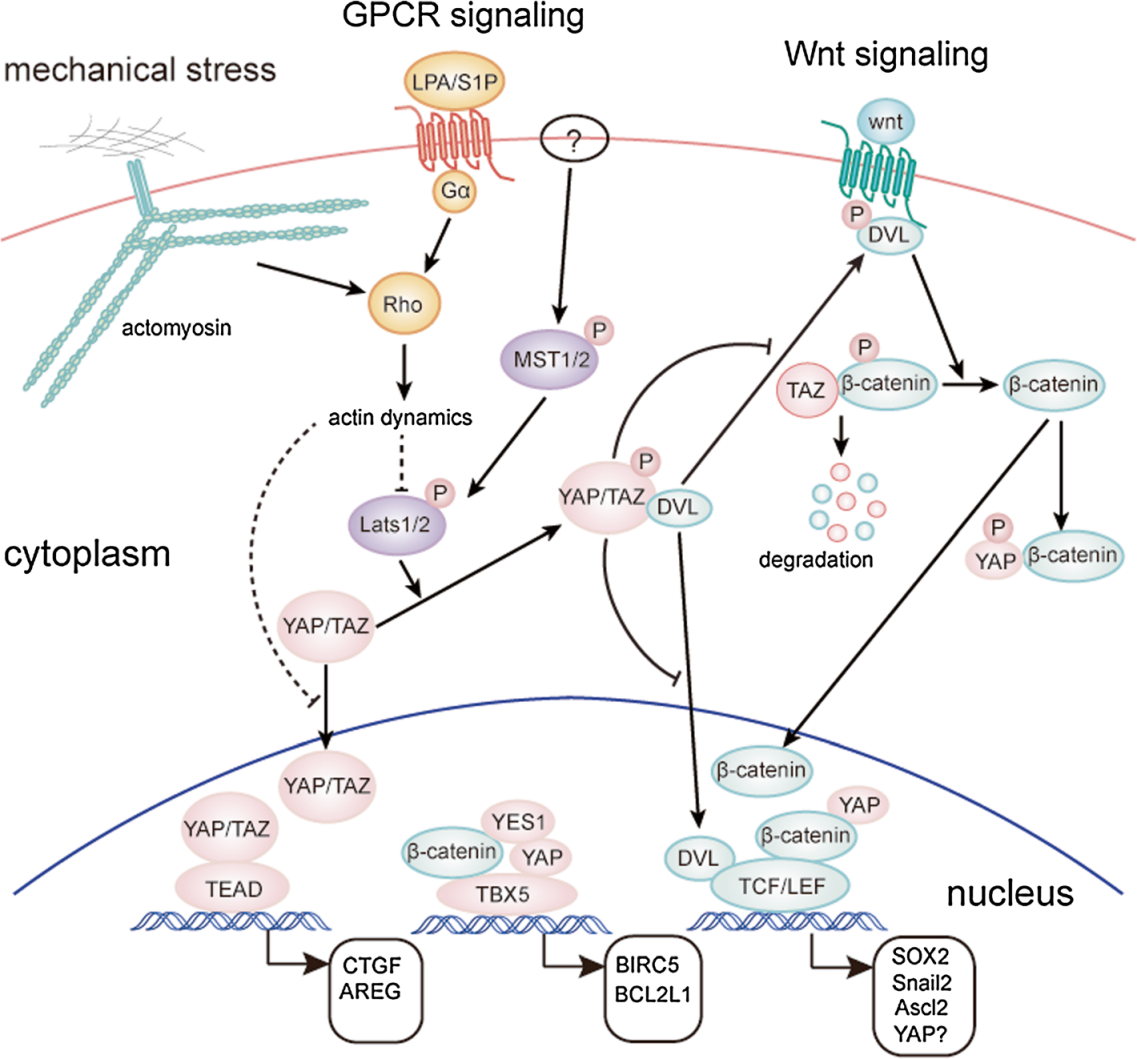
**YAP/TAZ are effectors of mechanical stress, GPCR signaling, and the Wnt signaling pathway.** Mechanisms of YAP and TAZ regulation by mechanical stress, GPCR signaling, and the Wnt pathway as well as YAP/TAZ as modulators of the Wnt pathway are shown. Arrowed or blunted ends indicate activation or inhibition, respectively. Dashed lines indicate unknown mechanisms.

The mechanism of YAP/TAZ regulation by cytoskeleton and mechanical stress is not completely understood. It was reported that knockdown of Lats1/2 is insufficient to rescue YAP/TAZ activity in cells cultured on soft matrix [25]. Nevertheless, in another report comparing cell attachment on stiff matrix or complete detachment, the Hippo pathway kinases Lats1/2 was found to be activated by cell detachment, also in a cytoskeleton-dependent manner [74]. And knockdown of Lats1/2 partially prevents mechanical stress-induced YAP phosphorylation and activation [74,75]. Thus it is possible that both Lats1/2-dependent and -independent mechanisms are involved in the YAP/TAZ regulation by mechanical stress (Figure 1). However, the factors and molecular mechanisms behind each possibility are unclear and await further characterization.

The biological relevance of YAP/TAZ-mediated response to mechanical stress has recently been demonstrated in breast cancer. Breast tumor is featured by its higher stiffness compared with normal mammary tissue, which is due to excessive deposition of collagen by stromal cells. Interestingly, it was found that YAP is activated in cancer-associated fibroblasts (CAFs), and its function is required for matrix stiffing [24]. Matrix stiffing further enhances YAP activity and thus forms a positive feedback loop creating a cancerous microenvironment. It was proposed that YAP conditioned the tumor microenvironment by stiffing the matrix to promote cancer cell invasion, although the role of YAP-induced secreted factors in this process was not formally excluded. Such activity in CAFs is observed only for YAP but not for TAZ. Nevertheless, only TAZ but not YAP was found to be associated with CSC maintenance and tumor progression in breast cancer [23]. Whether TAZ in epithelial cancer cells is activated by YAP-induced matrix stiffing awaits further confirmation. However, there seems to be an interesting cell-type specificity and job division between YAP and TAZ in the promotion of breast cancer.

### YAP/TAZ are effectors of GPCR signaling

Soluble molecules such as growth factors, morphogens, cytokines, and hormones serve as key factors initiating intracellular signaling pathways through association with and activation of their cognate receptors. In fact, most known signaling pathways involved in growth control in development and cancer are triggered by such extracellular cues. Despite the discovery of mechanical stress as a special upstream signal for YAP/TAZ, a soluble molecule as stimulant of the Hippo pathway was elusive. It was even unclear whether such a molecule exists. Nevertheless, two recent reports identified lysophosphatidic acid (LPA) and sphingosine-1-phosphophate (S1P), two related phospholipids in serum as potent activator of YAP/TAZ [26,27] (Figure 1). Following the initial observation of serum as a strong stimulant of YAP dephosphorylation and nuclear localization, both groups identified LPA and S1P as the active ingredients through a series of elegant biochemical analysis. Further experiments demonstrated that LPA and S1P activate YAP/TAZ by binding to their respective G-protein-coupled receptors (GPCRs) on cell surface and activation of the downstream heterotrimeric G proteins. Rho GTPase and remodeling of F-actin are known effectors of GPCR signaling and are also required for YAP/TAZ regulation by LPA and S1P. Thus the regulation of YAP/TAZ by mechanical stress and soluble factors converge on actin cytoskeleton remodeling. Activation of LPA and S1P receptors leads to inhibition of Lats1/2 kinase activity and knockdown of Lats1/2 partially blocks YAP phosphorylation induced by serum deprivation [26,27]. Thus Lats1/2 is at least partly responsible for YAP/TAZ regulation by LPA and S1P, although current evidence does not exclude the possibility of other mechanisms being involved in the process. However, Mst1/2 kinase activity is not regulated by these signals and ablation of Mst1/2 does not impair YAP/TAZ response to GPCR signaling [26,27]. This suggests that other molecules might be involved in regulation of the Hippo pathway kinases Lats1/2 in response to GPCR signaling.

It was later reported that thrombin, which activates protease-activated receptors, another GPCR, also stimulates YAP and TAZ activity [77]. In fact, it was found that YAP/TAZ is robustly regulated by many GPCRs and their cognate ligands [27]. In addition, YAP/TAZ activity could be either activated or inhibited depending on the G proteins coupled to the receptors. For example, activation of Gα_12/13_, Gα_q/11_, or Gα_i/o_ induces YAP/TAZ activity, whereas activation of Gα_s_ represses YAP/TAZ activity [27]. Therefore, YAP/TAZ seems to be a common target of GPCR signaling. It would then be interesting to determine whether these regulations on YAP/TAZ are all executed through a similar mechanism. And if not, what would be the individual mechanisms and for what kind of logic do these different signals converge on the regulation of YAP/TAZ.

GPCRs are the largest family of cell surface receptors mediating responses to a wide range of physiological signals and importantly, medicines [78]. Abnormal GPCR signaling is also involved in cancer development in many ways. Elevated expression of GCPRs such as PAR1 was found in high-grade breast cancers [79]. Furthermore, activating mutations of GPCRs have been found in several types of cancers such as melanomas and thyroid carcinomas [80,81]. In addition, activating mutations of Gα proteins have also been found in cancers, which is best exemplified by the remarkable Gα_q/11_ activating mutation rate of more than 80% in uveal melanomas [82,83]. It was reported that transgenic expression of LPA receptor 2 in mouse mammary glands induces activation of YAP/TAZ and massive overgrowth [27,84]. Thus, activation of YAP/TAZ might be involved in cancer induced by aberrant GPCR signaling. Such a possibility would need to be validated by experiments. Besides the pathological role of the GPCR-YAP/TAZ axis, it is also important to determine the role of this mechanism in organ size control in development and regeneration. Providing the large pool of circulating GPCR ligands including LPA and S1P, it would be important to identify the key player in organ size control in vivo.

### YAP/TAZ as effectors and modulators of Wnt signaling

Wnt is an important morphogen in development. In the canonical Wnt signaling pathway stimulation of Wnt receptors on cell surface results in disassembly of the β-catenin destruction complex. As a result, β-catenin accumulates in cell nuclei to stimulate expression of Wnt target genes [85]. In this way, β-catenin is a widely accepted effector of Wnt signaling pathway. Interestingly, a recent report discovered an unexpected role of TAZ as an effector of the Wnt pathway [28]. It was found that Wnt stimulation leads to stabilization of β-catenin as well as TAZ, but not YAP. TAZ physically interact with β-catenin and knockdown of β-catenin increases TAZ protein level and activity. It was proposed that β-catenin directs TAZ for co-degradation through SCF^β-TRCP^ mediated ubiquitination (Figure 1). Previously studies demonstrated that TAZ stability is regulated by phosphorylation of a C-terminal phospho-degron by the Hippo pathway and phosphorylation of an N-terminal degron by GSK3 [60,86]. Relationship between the three mechanisms has not been established yet. Noteworthy, it was also reported that Wnt/β-catenin promoted YAP protein level by activating YAP transcription [87]. Strikingly, gene expression profiling of mammary epithelial cells with knockdown of β-catenin or TAZ revealed that 74% of β-catenin target genes are also dependent on TAZ [28]. Such observations would suggest TAZ as a fundamental effector of the Wnt signaling pathway. However, the physiological or pathological relevance of this hypothesis is yet to be validated. Obviously, activation or inhibition of the Wnt pathway and the Hippo pathway results in very different phenotypes in animals suggesting differential roles in development. Furthermore, it was unclear whether TAZ is activated and plays a functional role in colon cancers, where abnormal activation of β-catenin plays a key role in tumorigenesis. In fact, another report published back-to-back with the above study identified YAP, but not TAZ as an essential survival factor for β-catenin-driven cancer cell lines [88]. In this study, 85 cancer cell lines were divided into β-catenin active and inactive groups based on TCF4 reporter activity. Further RNAi screen identified YAP as an essential gene for survival and anchorage-independent growth of β-catenin active cancer cell lines. However, suppression of TAZ expression did not affect the proliferation of these cell lines. Surprisingly, further experiments indicated that YAP supported the survival of β-catenin active cancer cells by partner with transcription factor TBX5 under the help of tyrosine kinase YES1. This transcriptional complex stimulates expression of genes such as BCL2L1 and BIRC5 to support cancer cell survival. However, the involvement of major YAP/TAZ target transcription factors, the TEAD family proteins, has not been excluded by experiments. Although TCF4 is the classical transcription factor partner of β-catenin, the report showed that YAP did not activate β-catenin on the TCF4 reporter. Nevertheless, in some other context such as mouse intestine and cardiomyocytes, activation of YAP due to inhibition of the Hippo pathway correlates with activation of β-catenin/TCF4 target genes [58,89]. Furthermore, YAP co-occupy gene promoters with β-catenin [89]. Therefore, the functional transcription factor partners of the YAP-β-catenin complex are not completely understood and could be context-dependent. Nevertheless, the above studies suggest a nuclear role of YAP/TAZ in modulating gene expression regulation and biological effects of the Wnt signaling pathway (Figure 1).

However, YAP/TAZ may also modulate the Wnt pathway through cytoplasmic mechanisms (Figure 1). In cultured cells, it was found that overexpression of YAP/TAZ inhibits β-catenin/TCF4 reporter activity and knockdown of YAP/TAZ activates it [90,91]. At the first look this would be contradictory to the nuclear function of YAP/TAZ in promoting beta-catenin activity. However, it was then demonstrated that cytoplasmic YAP and TAZ are the main force in inhibiting β-catenin. Cytoplasmic YAP may directly sequester β-catenin in the cytoplasm or cytoplasmic TAZ may sequester DVL2 impeding its activity to promote β-catenin accumulation in response to Wnt stimulation [90,91]. So that nuclear and cytoplasmic YAP/TAZ may have opposite roles in regulating β-catenin activity. The Hippo pathway is the best known mechanism for promoting cytoplasmic localization of YAP/TAZ. Thus the Hippo pathway may inhibit Wnt signaling through two distinct mechanisms, by repressing YAP/TAZ/β-catenin activity in cell nuclei and by promoting β-catenin cytoplasmic retention and possibly degradation.

**Figure 2:**
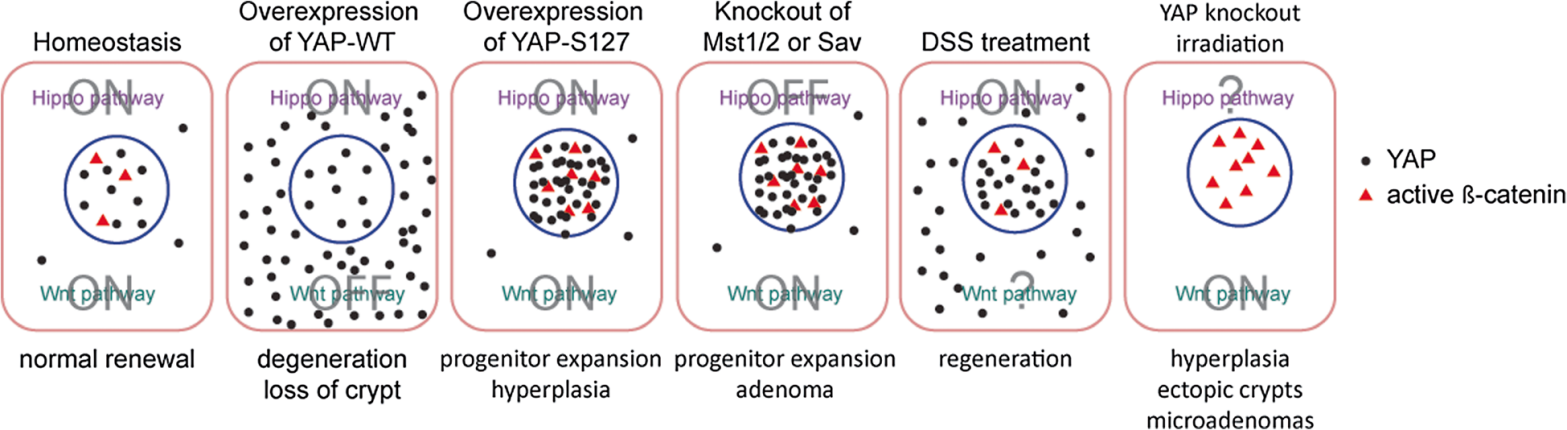
**Phenotypes of intestinal crypt cells in animal models concerning the cross-talk between the Hippo and Wnt pathways.** The protein level and subcellular localization of YAP in crypt cells of each mouse model are illustrated. The amount of transcriptionally active β-catenin in cell nucleus is also shown. However, for simplicity, the mechanisms are not reflected and the amount of transcriptionally active β-catenin does not suggest the change of total β-catenin protein level or subcellular localization.

Wnt pathway plays a key role in intestinal stem cell self-renewal and intestinal regeneration [85]. The function of YAP/TAZ in intestinal regeneration was also examined. Consistent with the role of YAP in promoting β-catenin activity in the nucleus and the growth promoting activity of itself, it was found that inactivation of YAP severely impairs dextran sodium sulfate (DSS)-induced intestinal regeneration, although YAP activity seems dispensable during development and normal homeostasis [18]. Noteworthy, the absence of phenotypes in YAP knockout intestines could be due to compensation by TAZ. During regeneration, YAP protein level was found to be elevated. Similarly, inactivation of Mst1 and Mst2 in mouse intestine also increases the protein level of YAP [58]. It was unclear whether the change of YAP protein level is related to Wnt signaling. However, further another group demonstrated that in irradiation-induced intestine regeneration model, loss of YAP leads to overexpansion of intestinal stem cells and development of microadenomas, which correlates with hyperactive Wnt signaling [19]. This finding would support an inhibitory role of YAP on Wnt signaling during regeneration although the underlying reason for differential YAP functions in the two intestine regeneration models is unknown. More surprisingly, although it was previously demonstrated that transgenic expression of an active YAP-S127A mutant strongly expands intestinal progenitors [39], tissue-specific expression of wild-type YAP leads to a progressive degeneration phenotype associated with loss of crypts and hypoactive Wnt signaling [19]. This would indicate that YAP, even the transgenicly expressed YAP is under inhibition by the Hippo pathway in intestinal epithelium, so that the Hippo pathway hypo-responsive S127A YAP mutant has different function from the wild-type protein. Furthermore, other than simply inactive, the extra abundant presumably cytoplasmic wild-type YAP protein plays some roles other than the nuclear YAP protein. The activity is possible as proposed to be the inhibition of β-catenin activity by restricting nuclear localization of DVL. It is worth noting that the intestinal phenotypes and altered Wnt signaling activity observed in wild-type YAP transgenic model and in YAP knockout model under irradiation-induced regeneration condition may be alternatively explained by loss or gain of Paneth cells due to the activity of YAP on cell differentiation [92]. Paneth cells serve as an important part of the stem cell niche and a major source of the Wnt ligand. Thus clarify the functions of YAP/TAZ in intestinal stem cells and their niche would be a crucial future direction. We summarized the complex phenotypes of intestinal crypt cells in reported mouse models concerning the cross-talk between the Hippo and Wnt pathways (Figure 2). Similar to the situation in intestinal regeneration, there are also contradictory reports on the correlation between YAP and colon cancer. YAP was reported to be both elevated in cancer and associated with a better prognosis [19,21,58]. A better understanding of the role of YAP in this disease would need a more quantitative assay for YAP level and localization and more high quality patient samples with disease progression data.

## Conclusions

YAP and TAZ transcription coactivators play key roles in organ size control, regeneration, and cancer development. Despite elucidation of their regulation by the Hippo pathway mediated phosphorylation and other mechanisms, the upstream physiological signals controlling YAP/TAZ activity has been elusive for a long time. Nevertheless, recent discoveries of YAP/TAZ as mediators of mechanical stress, GPCR signaling, and Wnt signaling open up the window to understand YAP/TAZ regulation under a complex physiological context in vivo with both physical and chemical properties. However, it is important to realize that in all three cases, key molecular mechanisms are still missing or are complicated by contradictory reports. For example, it is unclear how F-actin cytoskeleton regulates Lats1/2 kinase activity, which is important for YAP/TAZ regulation by both mechanical stress and GPCR signaling. Furthermore, identify of the possibly existing Lats1/2-independent mechanism of YAP/TAZ regulation by F-actin remodeling is yet to be uncovered. Moreover, the nuclear and cytoplasmic roles of YAP/TAZ in regulation of β-catenin activity need to be clarified and the mechanisms await further validation. In addition, the specificity and possibly differential roles of YAP and TAZ in mediating the above signals and in tissue regeneration and cancer also require more precise assessments. It is also interesting to understand how YAP/TAZ may serve to integrate different signals to mediate a proper response to the dynamic in vivo environment. For example, both mechanical stress and GPCR signaling input into regulation of Rho activity and thus affects YAP/TAZ activity. Therefore, it is interesting to know whether Rho serve as a checkpoint for both the physiological environment and the availability of chemical GPCR ligands to decide on cell proliferation and differentiation. Despite the existence of so many questions to be answered, the YAP/TAZ transcription coactivators are undoubtedly important mediators of physiological signals in regulation of organ size control, regeneration and tumorigenesis.

## Competing interests

The authors declare that they have no competing interests.

## Authors’ contributions

XG prepared the illustrations. BZ conceived the manuscript. XG and BZ drafted the manuscript. Both authors read and approved the final manuscript.
